# Protective Role of an Initial Low-Dose Septic Challenge against Lethal Sepsis in Neonatal Mice: A Pilot Study

**DOI:** 10.3390/jcm10245823

**Published:** 2021-12-13

**Authors:** Ruka Nakasone, Mariko Ashina, Takumi Kido, Harunori Miyauchi, Masafumi Saito, Shigeaki Inoue, Masakazu Shinohara, Kandai Nozu, Kazumichi Fujioka

**Affiliations:** 1 Department of Pediatrics, Kobe University Graduate School of Medicine, Kobe 650-0017, Japan; nakasone@med.kobe-u.ac.jp (R.N.); marikoa@med.kobe-u.ac.jp (M.A.); tkido@med.kobe-u.ac.jp (T.K.); nozu@med.kobe-u.ac.jp (K.N.); 2 Department of Pediatric Surgery, Kobe University Graduate School of Medicine, Kobe 650-0017, Japan; harukettamachine@gmail.com; 3 Department of Disaster and Emergency and Critical Care Medicine, Kobe University Graduate School of Medicine, Kobe 650-0017, Japan; masa9804chicco@gmail.com (M.S.); caf55000@gmail.com (S.I.); 4 Division of Epidemiology, Kobe University Graduate School of Medicine, Kobe 650-0017, Japan; mashino@med.kobe-u.ac.jp; 5 The Integrated Center for Mass Spectrometry, Kobe University Graduate School of Medicine, Kobe 650-0017, Japan

**Keywords:** neonatal sepsis, mouse model, trained immunity, cecal slurry, infection challenge, early immune modulation, inflammation

## Abstract

Neonatal sepsis is characterized by systemic bacterial invasion followed by a massive inflammatory response. At present, no therapeutic strategy has been found that significantly reduces the mortality of neonatal sepsis. We aimed to investigate the protective role of an initial low-dose septic challenge for the prevention of subsequent lethal sepsis in a mouse model. A stock cecal slurry (CS) solution was prepared from adult ceca. The LD83 (1.5 mg CS/g) was used for all animals. An initial challenge of normal saline (NS) or 0.5 mg CS/g (non-lethal dose) was administered at four days of age, then 1.5 mg CS/g was administered intraperitoneally at seven days of age (72 h post-initial challenge), and survival was monitored. Initial exposure to NS (*n* = 10) resulted in 90% mortality following exposure to the LD83 CS dose in contrast to an initial exposure to CS (*n* = 16), which significantly decreased mortality to 6% (*p <* 0.0001), reduced blood bacterial counts, attenuated inflammatory responses, and suppressed lipid mediators. Initial exposure to a non-lethal CS dose prior to exposure to a lethal CS dose significantly reduces sepsis mortality, a protective effect that might be mediated by modulating abnormal systemic inflammatory responses.

## 1. Introduction

Neonatal sepsis is a systemic infection caused by bacterial, viral, or fungal pathogens, and is accompanied by hemodynamic collapse and a variety of other clinical manifestations [1]. Despite recent advances in neonatal intensive care, sepsis is still one of the most common causes of neonatal mortality [2]. The mortality rate of neonatal sepsis is reported as 11–19%, and it is estimated that approximately three million infants are affected worldwide [3]. The morbidity and mortality of neonatal sepsis increases with decreasing birth weight and gestational age, especially for very preterm infants [4,5]. In addition, it is known that the prevalence of sepsis in newborns is higher than that in infants and older children [6]. The significant morbidity and mortality of sepsis in newborns are related to immune characteristics which are peculiar to neonates. Although newborns rely primarily on innate immunity, as the adaptive immune properties of newborns prioritize fetomaternal tolerance and contribute little to the protective immunity of the host [7], their innate immune response to infection is also underdeveloped, and they have decreased cytokine production and immature neutrophil and dendritic cell function compared to adults [8].

The standard treatment for neonatal sepsis is still antibiotic therapy, although immunomodulatory agents such as immunoglobulin, granulocyte colony-stimulating factor, granulocyte transfusion, and activated protein C have been studied as adjuvant treatments. In addition, in a neonatal mouse model of sepsis, the use of immunomodulatory agents, including Toll like receptor 4 (TLR4) or TLR7/8 agonists [7], aluminum salt-based adjuvants [9], and Bacille Calmette-Guérin (BCG) [10], have been investigated extensively. However, at present, no therapeutic strategy has been found that significantly reduces the mortality of neonatal sepsis [11]. Given the failure of post-septic treatment research to date, it would be beneficial to establish preventive methods in addition to elucidating the pathophysiology of neonatal sepsis. Some specific preventive approaches have been proposed to positively modify the immune response of neonates, including vaccination or limited exposure to non-infectious components of bacteria that are recognized by the innate immune system [12]. In recent years, it has been reported that certain infectious conditions and vaccinations induce a wide range of protective effects against other pathogens through the innate immune system, a characteristic known as “trained immunity” [13]. In 1986, Bistoni F et al. reported that intravenous administration of a non-lethal dose of *Candida Albicans* 7–14 days prior to lethal *Candida Albicans* infection provided protection from sepsis in an adult mouse model [14]. Intriguingly, this protective effect was also reproduced in recombination-activating gene 1 (Rag1)-deficient mice lacking functional T and B lymphocytes, and was found to act in a lymphocyte-independent monocyte-dependent manner [15]. In addition, epidemiological data suggested that the BCG (Bacille Calmette-Guérin) vaccine, a live attenuated vaccine against tuberculosis, has nonspecific protective effects against other infectious diseases [16]. Similarly, it was reported that the BCG vaccine induced nonspecific protection against lethal Candida infection in severe combined immunodeficiency (SCID) mice lacking functional T cells and B cells via activation of the innate immune system [17].

In adult intensive care, the concept of sepsis-induced immunosuppression has been widely accepted. Immunosuppression is caused by sepsis-induced immune cell apoptosis and by the direct impact of sepsis on immune cells, leading to secondary infections two to four weeks after the onset of sepsis and further exacerbating the infection [18]. In contrast, in neonatal intensive care, a large cohort study using the NICHD database found that very preterm infants with early onset sepsis (EOS, within three days of age) had a significantly lower risk of developing late-onset sepsis (LOS, after three days of age) or death [19]. Thus, it appears that there might be a fundamental difference in post-septic immunity between adults and newborns.

In addition, since the above-mentioned protective effect of the prior infection against secondary infection is thought to be based on activation of the innate immune response, it is not clear whether the same protective effect can be exerted in newborns with an underdeveloped innate immune response. Conventional sepsis research uses the cecal ligation and puncture method, which requires surgery and thus is mainly performed on adult animals. Wynn et al. have reported the use of the cecal slurry (CS) method [20], which does not require surgery and which makes sepsis research using neonatal mice possible [21]. Unlike other experimental neonatal sepsis models which use a single bacterial injection, the CS model exhibits polymicrobial sepsis and thus adequately replicates the pathology of neonatal sepsis [20]. In this study, we investigate the protective role of prior infection in neonatal sepsis by examining the effect of an initial low-dose septic challenge for the prevention of subsequent lethal sepsis using a neonatal sepsis mouse model created using the CS method.

## 2. Materials and Methods

### 2.1. Animals

Adult FVB/NJcl mouse breeders were purchased from CLEA Japan, Inc. (Tokyo, Japan) and provided a standard rodent diet and water *ad libitum*. All pups were kept with their mothers throughout the course of the study. The pups were randomized on an individual basis within each litter for each experiment. At least three different litters were used for survival experiments to eliminate any litter bias effects. For identification, the back or tail of each pup was labeled using a Sharpie marker as described previously [22]. This study was carried out in accordance with the ARRIVE guidelines and performed as approved by the Kobe University Institutional Animal Care and Use Committee (Protocol P180505).

### 2.2. CS Preparation

As described previously [21,22,23], a single stock CS solution was prepared from adult ceca and then stored at −80 °C in 1 mL aliquots until use. For examining bacterial viability, an aliquot of stock CS was thawed at room temperature, and 50 µL was plated onto 1.5% agar containing brain/heart infusion (BHI) broth [23]. Agar plates were then incubated at 37 °C for 24 h, and colony-forming units (CFU) were counted. For the entire study, the mean CFU count was 1.4 ± 0.3 × 10^5^ CFU/mL.

### 2.3. Sepsis Induction

**Figure 1:**
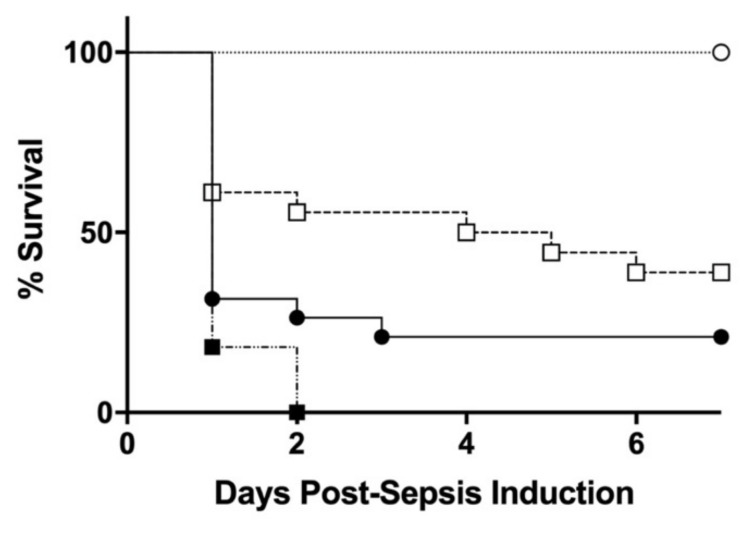
Dose dependent survival curves.

To induce sepsis, seven-day-old mouse pups that are immunologically equivalent to human term infants [24], were given various doses of CS intraperitoneally (IP) and then closely monitored daily for health and survival up to seven days, based on the method used in our previous studies [21,22]. Similar to that in a previous report, clear dose-dependent increase in mortality (0% for 0.5 mg/kg bodyweight (BW), 61% for 1.0 mg/kg BW, 83% for 1.5 mg/kg BW, and 100% for 2.0 mg/kg BW, *n* ≥ 10 in each group (Figure 1) was found [21,22]. For subsequent studies, a CS of 0.5 and 1.5 mg/kg BW were used for the initial low-dose septic challenge (non-lethal) and lethal septic induction, respectively.

Kaplan-Meier survival plots of seven-day-old WT pups after intraperitoneal (IP) injections of cecal slurry (CS) at various doses: 0.5 (○, *n* = 10), 1.0 (□, *n* = 18), 1.5 (●, *n* = 18), 2.0 (■, *n* = 11) mg/kg bodyweight (BW). A significant dose-dependent effect on mortality with LD_83_ of 1.5 mg/kg BW was found.

### 2.4. Initial Low-Dose Septic Challenge

To determine the efficacy of the initial low-dose septic challenge, we administered 0.5 mg/kg BW of CS (pretreatment, PTx) or vehicle (Veh) IP to four-day-old newborn pups and monitored BW and health for three days. On day seven (7-day-old), we induced sepsis by administering 1.5 mg/kg BW of CS IP to both groups and monitored survival and health for seven days, based on the approach in our previous studies.

### 2.5. Bacteriologic Examination

At 9 h post-lethal sepsis induction (i.e., seven days of life), pups were sacrificed by decapitation, and 60 to 80 µL of blood was immediately collected. Livers, lungs, and spleens were removed and rinsed with ice-cold 0.1 M KPO_4-_. Tissues were sonicated with the Ultrasonic Processor VCX-130 (Sonics & Materials, Inc., Newtown, CT, USA), and sonicates were diluted serially in PBS as follows: liver: undiluted, 1:10, and 1:100; lung: undiluted and 1:10; and spleen: undiluted, 1:10, 1:100, and 1:1000 [21,25]. Fifty microliters of samples were then streaked onto agar plates containing BHI broth as described previously [23]. Bacteria were grown at 37 °C for 24 h and quantified as CFU/mL as follows: dilution factor × CFUs × 1000 mL/mL divided by 50 mL [21].

### 2.6. Polymerase Chain Reaction (PCR) Arrays

At 6 h post-lethal sepsis induction, pups were sacrificed, and 5 mm × 5 mm × 1 mm pieces of liver were immediately placed in liquid nitrogen and stored at −80 °Cuntil use. Total RNA was extracted according to standard laboratory procedures using RNAeasy Mini Kit (Qiagen, Valencia, CA, USA). cDNA was synthesized by use of a RT2 First Strand Kit (Qiagen). PCR array kits (Qiagen) for mouse Innate & Adaptive immune Responses (catalog no. PAMM-052Z), which screens for 84 genes, were used. Real-time PCR was performed with RT2 Real-Time SYBR Green/ROX PCR Master Mix (Qiagen) on the Applied Biosystems (ABI) 7500 FAST Real-Time PCR system (Thermo Fisher Scientific, Waltham, MA, USA). Fold changes in gene expression levels in PTx or Veh over non-treated control (Cont) levels, and PTx over Veh levels were then calculated using the ΔΔCt method, as previously described [21,26].

### 2.7. Measurement of Lipid Mediators (LM)

The liver samples taken at 3 and 6 h (i.e., 7 days of life) post-lethal sepsis induction were homogenized in ice-cold methanol. Then, the deuterated internal standards were added to the samples (500 pg each) to facilitate quantification, as described previously. Samples underwent solid-phase extraction on C18 columns and were subjected to LC-MS/MS. The levels of targeted LMs were quantified by a multiple reaction monitoring method, and the LMs were identified using published criteria [22,27].

### 2.8. Statistical Analyses

Statistical analyses were performed using the log-rank test for survival curves, unpaired Student two-tailed *t*-test for PCR array and LMs analyses, and Mann-Whitney test or chi-square test for comparison of two groups. Differences were deemed statistically significant when *p* < 0.05.

## 3. Results

### 3.1. BW Changes Post-Initial Low Dose Septic Challenge

When comparing BW gain at 24 h and 72 h post-initial low dose septic challenge, the BW gains of the PTx groups were significantly lower than the BW gain of the non-septic Veh groups, respectively (24 h; 14.9 ± 7.7% vs. 22.1 ± 3.9%, *p* < 0.0001, 72 h; 55.4 ± 11.7% vs. 62.5 ± 9.0%, *p* < 0.01, *n* > 40 in each group).

### 3.2. Effect of Initial Septic Challenge on the Severity of Sepsis Mortality

**Figure 2:**
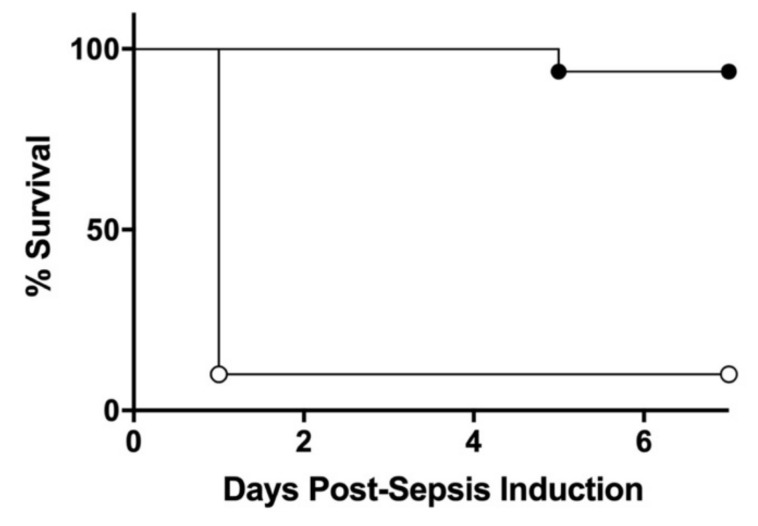
Survival curves of initial low-dose septic challenge and vehicle-treated pups.

The mortality rates of initially saline-treated pups (Veh; 90%, *n* = 10) were similar to the mortality of non-initially treated seven-day-old pups, as shown above (83%, *n* ≥ 10). The mortality rates of initially low-dose CS treated pups (PTx; 6%, *n* = 16) were significantly lower than those of Veh groups (*p* < 0.0001, Figure 2).

Kaplan-Meier survival plots of seven-day-old mouse pups pretreated with 0.5 mg/kg BW of CS (pretreatment, PTx: ●, *n* = 16) or equivalent volume of vehicle (Veh: ○, *n* = 10) IP 72 h prior to IP injection of 1.5 mg/kg BW of CS. The survival rate of PTx pups was significantly higher than those of Veh pups (*p <* 0.0001, log-rank test).

### 3.3. Organ Bacterial Colonization

**Figure 3:**
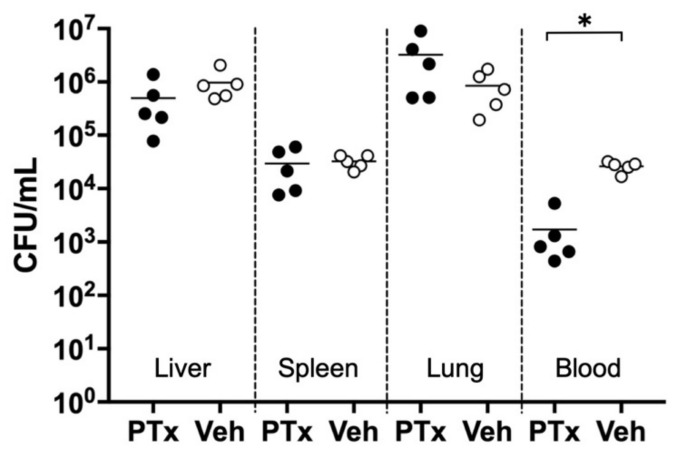
CFU counts in organ sonicates prepared 9 h post-sepsis induction.

To investigate the effect of initial low-dose septic challenge on bacterial colonization, we compared CFU counts in each organ 9 h post-lethal sepsis induction between PTx and Veh pups. We found no significant differences in CFU counts (expressed as CFU/mL) in the liver (PTx: 4.96 ± 5.20 × 10^5^ vs. Veh: 9.75 ± 6.40 × 10^5^, *n* = 5 in each group, *p* = 0.23), lung (2.95 ± 2.39 × 10^4^ vs. 3.24 ± 0.91 × 10^4^, *n* = 5 in each group, *p* = 0.81), and spleen (3.27 ± 3.56 × 10^6^ vs. 0.86 ± 0.64 × 10^6^, *n* = 5 in each group, *p* = 0.17). However, CFU counts in the blood of PTx pups were significantly less than those of Veh pups (1.71 ± 2.03 × 10^3^ vs. 2.62 ± 0.57 × 10^4^, *n* = 5 in each group, *p* < 0.0001, Figure 3).

Serial dilutions were made of organ sonicates prepared from pretreatment (PTx: ●, *n* = 5 for each organ) or vehicle-treated (Veh: ○, *n* = 5 for each organ) pups 9 h post-sepsis induction, and then incubated on BHI agar plates at 37 °C for 24 h. There were no significant differences in CFU counts in the liver, spleen, and lung between the two groups, whereas CFU counts in the blood of PTx pups decreased significantly in comparison with those of Veh pups (* *p <* 0.0001).

### 3.4. Effect of Initial Septic Challenge on Expression Profiles of Genes Involved in Innate and Adaptive Immunity Post-Lethal Sepsis Induction

To determine the expression levels of genes involved in innate and adaptive immune responses, we isolated RNA from livers harvested 6 h post-lethal sepsis induction. Using RT-PCR arrays, we found that of the 84 genes screened by the kit, five gene transcripts significantly increased in livers of Veh-CS treated (Veh) pups compared to non-septic Veh-Veh treated (Cont) pups (fold change > 4.0, *p* < 0.05, Table 1). The increased genes were cytokines (IL-10, IL-1a, IL-1b, IL-6) and an immune response related gene (M × 1). In contrast, only one gene transcript significantly increased and two gene transcripts significantly decreased in livers of CS-CS treated (PTx) pups over non-septic Cont groups (fold change > 4.0, *p* < 0.05, Table 1). The increased gene (Rorc) and decreased genes (Cd14, IL-1r1) were immune response related genes. 

We then compared the gene expression profiles of PTx and Veh pups 6 h post-lethal sepsis induction. We found that most of the genes upregulated post-sepsis induction were down-regulated after the initial septic challenge. In particular, five genes related to immune response (Cd14, C5aR1, IL1r1, Mx1, Irf7) were significantly down-regulated (>4-fold, *p* < 0.05, Table 2).

### 3.5. Effect of Initial Septic Challenge on LM Post-Sepsis Induction

At 3 h post-lethal sepsis induction, the livers of the PTx group contained one arachidonic acid (AA)-derived LM (15deoxy-d12,14 PGJ_2_), which was significantly decreased compared with Veh group, whereas, at 6 h post-lethal sepsis induction, four eicosapentaenoic acid (EPA)-derived LMs (12-HEPE, 15-HEPE, 18-HEPE, EPA), five docosahexaenoic acid (DHA)-derived LMs (4-HDHA, 7-HDHA, 14-HDHA, 17-HDHA, DHA), and six AA-derived LMs (5-HETE, 12-HETE, 15-HETE, AA) were significantly decreased in livers of PTx groups, compared with Veh groups (Table 3).

## 4. Discussion

In this study, we found that the initial low-dose septic challenge attenuated sepsis severity in a non-surgical neonatal sepsis mouse model. Furthermore, levels of several inflammatory genes and LMs 6 h post-lethal sepsis induction were suppressed by pretreatment.

In our model, intraperitoneal administration of a low non-lethal dose of CS three days prior to lethal sepsis induction led to improvements in the mortality rate, despite reduced body weight gain post-initial septic challenge. The non-surgical neonatal mouse model used in this study induces polymicrobial sepsis by intraperitoneal administration of CS in five to seven day-old newborn pups, and was first reported by Wynn et al [20]. To date, several sepsis treatment strategies have been studied using this model. Their group reported the protective effect of intraperitoneal injection of the TLR4 or TLR7/8 agonist 24 h prior to the initiation of sepsis, resulting in increased survival and reduced bacteremia via enhanced peritoneal neutrophil recruitment [7]. In addition, they reported that pretreatment with aluminum salts-based adjuvants, which are used in pediatric vaccines, 24 h prior to sepsis induction improved survival in both wild-type and T and B cell-deficient neonatal mice, suggesting stimulation of multiple innate immune cell functions [9]. Similar to our models, Brook et al. subcutaneously administered 50 µL of BCG vaccine to four to five day-old neonatal mice, followed three days later by intraperitoneally injected CS to induce polymicrobial sepsis, and reported a significant increase in survival associated with induced granulocyte colony-stimulating factor within hours of administration [10]. From the perspective of direct activation of immune cells, Bolognese et al. have reported a protective effect of activating Invariant Natural Killer T (iNKT) cells in neonatal sepsis. In their study, intraperitoneal injection of the iNKT stimulator KRN7000 30 h prior to sepsis induction resulted in better outcomes in inflammation, lung injury, and survival by increasing systemic levels of TGF-β1 [28]. All these studies have illustrated that preconditioning the immune response is effective in sepsis protection, even in neonatal mice. However, our report demonstrates that an initial low-dose septic challenge has a protective effect against lethal sepsis and most closely replicates the physiology of the clinical condition.

We observed an increase in gene expression profiles for cytokines and other genes related to immune response in Veh pups compared to non-septic Cont pups. Interestingly, these increased genes were significantly down-regulated in the livers of PTx pups compared to Veh pups, and associated with increased survival. In neonatal sepsis, systemic inflammatory immune responses are closely associated with increased morbidity and mortality despite their role in reducing microbial invasion [29,30]. In an LPS-induced sepsis model, administration of 10 g/kg LPS to mice of different ages (one day, seven days, two weeks, and 10 weeks), a significant age-dependent increase in sepsis survival, and a decrease in serum inflammatory cytokines 6 h post-sepsis induction was reported [30]. The high mortality of neonatal sepsis might be due to excessive systemic inflammatory responses, and the protective effect of an initial low-dose septic challenge that we observed may result from modulation of these responses. In addition, we observed that two LMs were significantly suppressed at 3 h, and 13 LMs were significantly suppressed at 6 h post-sepsis induction in PTx pups compared to Veh pups. In our previous study reporting the protective effect of recombinant human thrombomodulin (rhTM) in a preterm sepsis mouse model, five LMs were significantly suppressed in the rhTM-treated pups 3 h post-sepsis induction, and an improved survival rate was observed [22]. We proposed that the suppression of LM elevation during sepsis might be associated with the favorable results in the PTx groups that were seen in this study.

For humans, in the area of adult intensive care, it is widely known that chronic immunosuppression is prolonged in sepsis survivors’ post-intensive care. This condition is known as persistent inflammation, immunosuppression, and catabolism syndrome (PICS) [31]. PICS has been shown to affect long-term outcomes such as secondary nosocomial infections, prolonged hospitalization, and impaired reintegration. This immunosuppressive state is mediated by a post-septic increase in immature polynuclear leukocytes, bone marrow-derived suppressor cells, and M2 macrophages, and by a post-septic decrease in T, B, and dendritic cells via apoptosis [32]. In contrast, in the area of neonatal intensive care, Wynn et al. reported from their retrospective analysis of 34,396 VLBW infants that the risk of developing LOS was shorter in infants with BW of 401–750 g who had suffered from EOS (relative risk (RR): 0.80 [0.64–0.99]), although no difference in LOS risk was found overall for infants with versus without EOS [RR: 0.88 (0.75–1.02)]. They speculate that this mechanism might be due to an absence of immunoparalysis in neonates or that an early immune stimulus may transform the host defense status of the preterm infant from a relative state of tolerance to a level of competence in the extrauterine environment [19]. Consistent with their results, three RCTs on the effects of BCG vaccination immediately after birth on low birth weight infants (BW < 2500 g) have been conducted between 2002 and 2013, and meta-analysis of these trials revealed that BCG vaccination immediately after birth reduced overall mortality 28 days after birth by 38% [33] and in-hospital sepsis mortality by 54% [34]. These findings suggest that training the innate immune system by BCG vaccination has a protective effect against nonspecific pathogens. Our observations appear to support a similar mechanism for low-dose septic challenge.

This study had some limitations. First, we could only analyze gene expression profiles and LM dynamics at early time points post-sepsis induction, since most of the pups in the Veh group died within 24 h after induction of sepsis. In the future, it would be preferable to analyze hemodynamics and immunoinflammatory responses at later time points using a model with a lower sepsis severity. Second, since this study was conducted using neonatal mice, it was difficult to collect an adequate quantity of tissue and blood, and it was therefore not possible perform a detailed immune cell analysis. To elucidate the mechanism of immune training in this model, the characteristics and functions of immune cells in the peripheral blood, spleen, and peritoneal lavage fluid should be examined using larger animals. Future studies should assess the immune response (B and T cells, and inflammatory mediators) to assess the components of the complex immune response to early infection that may contribute to a future reduction in sepsis mortality. Third, although our model uses essentially the same type of bacteria for the initial low-dose and the following severe sepsis induction, early and late onset sepsis in preterm infants is typically caused by different species of bacteria. In addition, we did not administer antibiotics in this model, which is routine practice in the clinical setting, because the injection of antibiotics is too invasive for the neonatal pups used in our study. Thus, although the data generated from these experiments may be useful for future hypothesis generation and testing, the concept still requires further validation in a more clinically relevant model. Finally, we have not yet investigated how long the protective effect of initial low-dose septic challenge lasts; thus, a future study should further extend the period from initial septic challenge to the induction to lethal sepsis.

## 5. Conclusions

An initial low-dose septic challenge attenuated sepsis severity in a non-surgical neonatal sepsis mouse model, which may be mediated by control of the abnormal systemic inflammatory response. Early immune modulation, similar to the effects induced by the infection challenge used in this model, should be explored further as a novel treatment strategy for late-onset sepsis in preterm infants.

## Figures and Tables

**Table 1:** Fold changes in expression levels of immune genes in the initial septic challenge and vehicle groups.

	Veh-CS Treated (Veh) vs. Veh-Veh Treated Control (Cont)		CS-CS Treated (PTx) vs. Veh-Veh Treated Control (Cont)	
Gene Symbol	Fold Change	*p*	Fold Change	*p*
*Cd14*	1.63	0.039	−58.35	0.029
*Il10*	5.38	0.002	−2.15	0.623
*Il1a*	6.31	0.036	1.21	0.592
*Il1b*	8.09	0.016	−1.57	0.540
*Il1r1*	1.60	0.020	−7.15	0.036
*Il6*	9.90	0.002	1.43	0.518
*Mx1*	15.81	0.023	1.01	0.853
*Rorc*	1.87	0.005	4.87	0.015

Fold change is calculated as expression levels of treated groups (*n* = 3 in each group) over that of Veh-treated (Cont) pups (*n* = 3). PTx; initial septic challenge group, Veh; vehicle group, Cont; non-septic control group.

**Table 2:** Fold changes in expression levels of immune genes in the initial septic challenge group.

	CS-CS Treated (PTx) over Veh-CS Treated (Veh)	
Gene Symbol	Fold Change	*p*
*Cd14*	−95.29	0.009
*Mx1*	−15.64	0.024
*Il1b*	−12.68	0.333
*Il10*	−11.58	0.226
*Il1r1*	−11.43	0.002
*Il6*	−6.94	0.096
*Il1a*	−5.20	0.094
*Irf7*	−4.55	0.020
*C5ar1*	−4.4	0.006
*Rorc*	2.6	0.032

Fold change was calculated as the expression levels of PTx groups (*n* = 3) compared to those of the Veh group (*n* = 3). CS, cecal slurry; PTx, initial septic challenge group; Veh, vehicle group.

**Table 3:** Effect of initial septic challenge on LM parameters.

LM (pg/mg Tissue).		Post 3 h		Post 6 h	
		Veh (*n* = 4)	PTx (*n* = 4)	Veh (*n* = 4)	PTx (*n* = 4)
EPA	Resolvin E3	23.5 ± 6.6	15.9 ± 1.1	8.2 ± 2.6	3.7 ± 0.6
	5-HEPE	1.7 ± 0.6	1.8 ± 0.1	0.9 ± 0.2	0.4 ± 0.0
	12-HEPE	1.5 ± 0.6	1.5 ± 0.1	1.5 ± 0.3	0.6 ± 0.2 *
	15-HEPE	1.2 ± 0.3	1.3 ± 0.2	0.6 ± 0.1	0.2 ± 0.0 *
	18-HEPE	2.1 ± 0.6	2.3 ± 0.4	1.0 ± 0.2	0.5 ± 0.0 *
	EPA	149.8 ± 51.9	184.1 ± 33.9	100.7 ± 12.9	60.6 ± 9.2 *
DHA	4-HDHA	3.5 ± 0.8	3.7 ± 0.5	5.2 ± 0.5	2.3 ± 0.4 **
	7-HDHA	1.0 ± 0.2	1.3 ± 0.2	0.9 ± 0.1	0.5 ± 0.1 **
	14-HDHA	2.7 ± 0.6	3.1 ± 0.4	3.4 ± 0.4	1.5 ± 0.3 *
	17-HDHA	5.4 ± 1.4	6.2 ± 0.8	5.3 ± 0.8	2.2 ± 0.2 **
	DHA	474 ± 109.5	620.3 ± 58.2	706.6 ± 56.3	433.2 ± 94.9 *
AA	PGE_2_	15.7 ± 3.5	18.5 ± 2.3	27.8 ± 9.2	25.1 ± 4.8
	PGD_2_	133.8 ± 29.6	142.3 ± 56.5	212.1 ± 55.0	171.3 ± 16.7
	15deoxy-d12,14 PGJ_2_	147.7 ± 30.3	67.3 ± 11.4 *	119.7 ± 18.0	111.1 ± 30.1
	PGF_2a_	91.4 ± 29.6	34.9 ± 8.5	134.9 ± 91.7	24.7 ± 3.2
	TxB_2_	30.7 ± 4.2	23.2 ± 9.1	38.6 ± 11.8	40.9 ± 9.5
	12S-HHT	9.8 ± 3.7	7.2 ± 1.5	13.8 ± 6.1	5.0 ± 0.9
	LTB_4_	0.1 ± 0.1	0.3 ± 0.1	0.2 ± 0.0	0.1 ± 0.0
	Lipoxin B_4_	70.7 ± 22.0	49.8 ± 13.6	31.2 ± 8.8	13.0 ± 2.8
	5-HETE	4.7 ± 1.3	5.3 ± 0.4	6.0 ± 0.8	2.3 ± 0.3 **
	12-HETE	7.1 ± 2.0	7.4 ± 0.6	13.0 ± 1.5	4.8 ± 1.3 **
	15-HETE	7.6 ± 2.1	9.4 ± 1.3	7.6 ± 0.7	3.8 ± 0.4 **
	AA	547.6 ± 156.0	598.1 ± 8.9	725.1 ± 64.9	364.8 ± 57.5 **

Results are expressed as mean ± standard error of mean. * *p* < 0.05 vs. Veh, ** *p* < 0.01 vs. Veh. PTx, initial septic challenge group; Veh, vehicle; EPA, eicosapentaenoic acid; DHA, docosahexaenoic acid; AA, arachidonic acid; LM, lipid mediators.

## Data Availability

All relevant data are contained within this manuscript.

## References

[1] Shane A.L., Sanchez P.J., Stoll B.J. (2017). Neonatal sepsis. Lancet.

[2] Liu L., Oza S., Hogan D., Perin J., Rudan I., Lawn J.E., Cousens S., Mathers C., Black R.E. (2015). Global, regional, and national causes of child mortality in 2000–2013, with projections to inform post-2015 priorities: An updated systematic analysis. Lancet.

[3] Fleischmann-Struzek C., Goldfarb D.M., Schlattmann P., Schlapbach L.J., Reinhart K., Kissoon N. (2018). The global burden of paediatric and neonatal sepsis: A systematic review. Lancet Respir. Med..

[4] Stoll B.J., Hansen N.I., Bell E.F., Shankaran S., Laptook A.R., Walsh M.C., Hale E.C., Newman N.S., Schibler K., Carlo W.A. (2010). Neonatal outcomes of extremely preterm infants from the NICHD Neonatal Research Network. Pediatrics.

[5] Stoll B.J., Hansen N.I., Sanchez P.J., Faix R.G., Poindexter B.B., Van Meurs K.P., Bizzarro M.J., Goldberg R.N., Frantz I.D., Hale E.C. (2011). Early onset neonatal sepsis: The burden of group B Streptococcal and *E. coli* disease continues. Pediatrics.

[6] Hartman M.E., Linde-Zwirble W.T., Angus D.C., Watson R.S. (2013). Trends in the epidemiology of pediatric severe sepsis. Pediatr. Crit. Care Med..

[7] Wynn J.L., Scumpia P.O., Winfield R.D., Delano M.J., Kelly-Scumpia K., Barker T., Ungaro R., Levy O., Moldawer L.L. (2008). Defective innate immunity predisposes murine neonates to poor sepsis outcome but is reversed by TLR agonists. Blood.

[8] Raymond S.L., Stortz J.A., Mira J.C., Larson S.D., Wynn J.L., Moldawer L.L. (2017). Immunological Defects in Neonatal Sepsis and Potential Therapeutic Approaches. Front. Pediatr..

[9] Rincon J.C., Cuenca A.L., Raymond S.L., Mathias B., Nacionales D.C., Ungaro R., Efron P.A., Wynn J.L., Moldawer L.L., Larson S.D. (2018). Adjuvant pretreatment with alum protects neonatal mice in sepsis through myeloid cell activation. Clin. Exp. Immunol..

[10] Brook B., Harbeson D.J., Shannon C.P., Cai B., He D., Ben-Othman R., Francis F., Huang J., Varankovich N., Liu A. (2020). BCG vaccination-induced emergency granulopoiesis provides rapid protection from neonatal sepsis. Sci. Transl. Med..

[11] Wynn J.L., Neu J., Moldawer L.L., Levy O. (2009). Potential of immunomodulatory agents for prevention and treatment of neonatal sepsis. J. Perinatol..

[12] Sadarangani M., Kollmann T., Bjornson G., Heath P., Clarke E., Marchant A., Levy O., Leuridan E., Ulloa-Gutierrez R., Cutland C.L. (2021). The Fifth International Neonatal and Maternal Immunization Symposium (INMIS 2019): Securing Protection for the Next Generation. mSphere.

[13] Netea M.G., Dominguez-Andres J., Barreiro L.B., Chavakis T., Divangahi M., Fuchs E., Joosten L.A.B., van der Meer J.W.M., Mhlanga M.M., Mulder W.J.M. (2020). Defining trained immunity and its role in health and disease. Nat. Rev. Immunol..

[14] Bistoni F., Vecchiarelli A., Cenci E., Puccetti P., Marconi P., Cassone A. (1986). Evidence for macrophage-mediated protection against lethal Candida albicans infection. Infect. Immun..

[15] Quintin J., Saeed S., Martens J.H.A., Giamarellos-Bourboulis E.J., Ifrim D.C., Logie C., Jacobs L., Jansen T., Kullberg B.J., Wijmenga C. (2012). Candida albicans infection affords protection against reinfection via functional reprogramming of monocytes. Cell Host. Microb..

[16] Benn C.S., Netea M.G., Selin L.K., Aaby P. (2013). A small jab—A big effect: Nonspecific immunomodulation by vaccines. Trends Immunol..

[17] Kleinnijenhuis J., Quintin J., Preijers F., Joosten L.A., Ifrim D.C., Saeed S., Jacobs C., van Loenhout J., de Jong D., Stunnenberg H.G. (2012). Bacille Calmette-Guerin induces NOD2-dependent nonspecific protection from reinfection via epigenetic reprogramming of monocytes. Proc. Natl. Acad. Sci. USA.

[18] Hotchkiss R.S., Monneret G., Payen D. (2013). Sepsis-induced immunosuppression: From cellular dysfunctions to immunotherapy. Nat. Rev. Immunol..

[19] Wynn J.L., Hansen N.I., Das A., Cotten C.M., Goldberg R.N., Sanchez P.J., Bell E.F., Van Meurs K.P., Carlo W.A., Laptook A.R. (2013). Early sepsis does not increase the risk of late sepsis in very low birth weight neonates. J. Pediatr..

[20] Wynn J.L., Scumpia P.O., Delano M.J., O’Malley K.A., Ungaro R., Abouhamze A., Moldawer L.L. (2007). Increased mortality and altered immunity in neonatal sepsis produced by generalized peritonitis. Shock.

[21] Fujioka K., Kalish F., Zhao H., Lu S., Wong S., Wong R.J., Stevenson D.K. (2017). Induction of Heme Oxygenase-1 Attenuates the Severity of Sepsis in a Non-Surgical Preterm Mouse Model. Shock.

[22] Ashina M., Fujioka K., Nishida K., Okubo S., Ikuta T., Shinohara M., Iijima K. (2020). Recombinant human thrombomodulin attenuated sepsis severity in a non-surgical preterm mouse model. Sci. Rep..

[23] Starr M.E., Steele A.M., Saito M., Hacker B.J., Evers B.M., Saito H. (2014). A new cecal slurry preparation protocol with improved long-term reproducibility for animal models of sepsis. PLoS ONE.

[24] Adkins B., Leclerc C., Marshall-Clarke S. (2004). Neonatal adaptive immunity comes of age. Nat. Rev. Immunol..

[25] Kronforst K.D., Mancuso C.J., Pettengill M., Ninkovic J., Power Coombs M.R., Stevens C., Otto M., Mallard C., Wang X., Goldmann D. (2012). A neonatal model of intravenous Staphylococcus epidermidis infection in mice <24 h old enables characterization of early innate immune responses. PLoS ONE.

[26] Fujioka K., Kalish F., Zhao H., Wong R.J., Stevenson D.K. (2018). Heme oxygenase-1 deficiency promotes severity of sepsis in a non-surgical preterm mouse model. Pediatr. Res..

[27] Colas R.A., Shinohara M., Dalli J., Chiang N., Serhan C.N. (2014). Identification and signature profiles for pro-resolving and inflammatory lipid mediators in human tissue. Am. J. Physiol. Cell Physiol..

[28] Bolognese A.C., Yang W.L., Hansen L.W., Sharma A., Nicastro J.M., Coppa G.F., Wang P. (2018). Activation of Invariant Natural Killer T Cells Redirects the Inflammatory Response in Neonatal Sepsis. Front. Immunol..

[29] Speer E.M., Diago-Navarro E., Ozog L.S., Raheel M., Levy O., Fries B.C. (2020). A Neonatal Murine Escherichia coli Sepsis Model Demonstrates That Adjunctive Pentoxifylline Enhances the Ratio of Anti- vs. Pro-inflammatory Cytokines in Blood and Organ Tissues. Front. Immunol..

[30] Zhao J., Kim K.D., Yang X., Auh S., Fu Y.X., Tang H. (2008). Hyper innate responses in neonates lead to increased morbidity and mortality after infection. Proc. Natl. Acad. Sci. USA.

[31] Gentile L.F., Cuenca A.G., Efron P.A., Ang D., Bihorac A., McKinley B.A., Moldawer L.L., Moore F.A. (2012). Persistent inflammation and immunosuppression: A common syndrome and new horizon for surgical intensive care. J. Trauma Acute Care Surg..

[32] Hotchkiss R.S., Moldawer L.L., Opal S.M., Reinhart K., Turnbull I.R., Vincent J.L. (2016). Sepsis and septic shock. Nat. Rev. Dis. Primers.

[33] Biering-Sorensen S., Aaby P., Lund N., Monteiro I., Jensen K.J., Eriksen H.B., Schaltz-Buchholzer F., Jorgensen A.S.P., Rodrigues A., Fisker A.B. (2017). Early BCG-Denmark and Neonatal Mortality Among Infants Weighing <2500 g: A Randomized Controlled Trial. Clin. Infect. Dis..

[34] Schaltz-Buchholzer F., Biering-Sorensen S., Lund N., Monteiro I., Umbasse P., Fisker A.B., Andersen A., Rodrigues A., Aaby P., Benn C.S. (2019). Early BCG Vaccination, Hospitalizations, and Hospital Deaths: Analysis of a Secondary Outcome in 3 Randomized Trials from Guinea-Bissau. J. Infect. Dis..

